# Position Matters: Fluorescent Positional Isomers for Reliable Multichannel Encryption Devices

**DOI:** 10.1002/chem.202103441

**Published:** 2021-10-29

**Authors:** Yuxin Liu, Peter H. Seeberger, Nabyl Merbouh, Felix F. Loeffler

**Affiliations:** ^1^ Department of Biomolecular Systems Max Planck Institute of Colloids and Interfaces Am Muehlenberg 1 14476 Potsdam Germany; ^2^ Department of Chemistry Simon Fraser University Burnaby, B.C. Canada; ^3^ Institute of Chemistry and Biochemistry Free University of Berlin 14195 Berlin Germany

**Keywords:** acid response, encryption, fluorescence, multichannel, positional isomers

## Abstract

Fluorescence signals have been widely used in information encryption for a few decades, but still suffer from limited reliability. Here, reversible multichannel fluorescent devices with encrypted information were constructed, based on two fluorescent positional isomers of a diphenylquinoxaline derivative. Possessing the same core fluorescent group and acid‐/pH‐responsive mechanism, the two isomers showed different fluorescence colors in an acidic environment; this allowed us to realize stepwise encryption of information in orthogonal fluorescence channels. Because the protonation was reversible, the revealed information could be re‐encrypted simply by heating. This approach highlights the value of positional isomers to build multichannel encryption devices, improving their reliability on the molecular level.

## Graphical abstract

**The asymmetry** in the electron distribution of positional isomers of quinoxaline derivatives results in distinguishable acid‐/pH‐responsive emissions; this can function as the basis for enabling the construction of multichannel encryption devices. The reliability of encryption is improved by the minimized compositional difference and responsive mechanism on the molecular level.

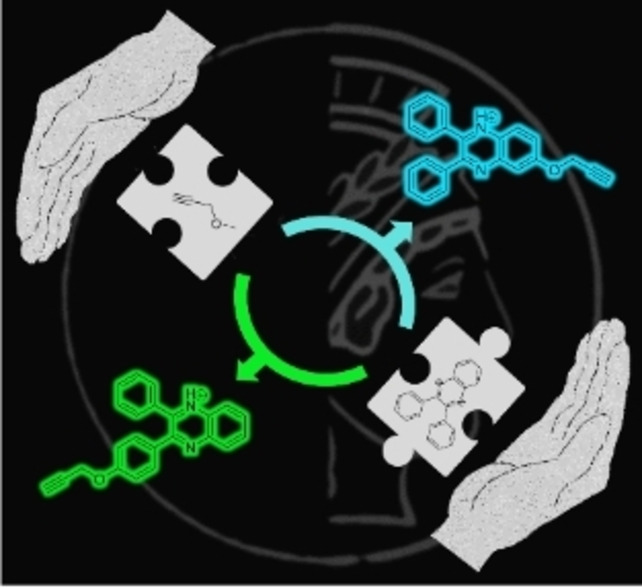

Chemistry‐based information encryption has been studied and applied for hundreds of years and is still useful in modern society.^[1]^ Among all the methods, fluorescent encryption has gathered much attention in recent decades due to its advantages in visualization and timeliness.^[2]^ By using physically or chemically responsive fluorophores, the information presented by fluorescent signals can be readily decrypted and re‐encrypted, which reduces the accidental release of sensitive information.^[3]^

However, the fluorescent encryption is still at potential risk of brute‐force cracking. As all information is encrypted and stored in one single fluorescence channel, a repeated matter of trial and error will eventually lead to the key.^[4]^ This issue can be effectively addressed by introducing multiple fluorescence channels into an encryption device and manipulating the read‐out signals, but it brings another breakable chain for encryption.^[5]^ The construction of a multichannel fluorescent device requires multiple responsive fluorophores to obtain separable fluorescence channels and, typically, these fluorophores have different chemical structures or functional groups.^[6]^ In this case, the specificity of the key is reduced, as the recognition of the fluorophore types by chemical reactivity or responsive mechanism is also applicable to read‐out information (e. g., binary code) without decryption of fluorescence signals. Therefore, a group of fluorophores, with minimal difference in their chemical composition, functional groups, and even structure, would be of great interest in the improvement of key specificity and, hence, encryption safety.

Aiming for this goal, two fluorescent positional isomers, 2‐phenyl‐3‐(4‐(prop‐2‐yn‐1‐yloxy)phenyl)quinoxaline (i. e., Alk‐DQ‐1) and 2,3‐diphenyl‐6‐(prop‐2‐yn‐1‐yloxy)quinoxaline (i. e., Alk‐DQ‐2), were designed and synthesized (Figures S1 and S2 in the Supporting Information). The quinoxaline structure conveyed acid‐responsive fluorescent properties, while the alkyne moiety allowed the connection to an azido‐modified glass slides in a Cu^I^‐catalyzed click reaction to construct patterns (Scheme 1). Like all quinoxaline derivatives, the two nitrogen atoms can be protonated to its cationic form, which was confirmed by the downfield‐shifted aromatic hydrogens in the ^1^H NMR spectra (Figures S3 and S4).^[7]^ By evaluating the energies of different possible cations, the one with the protonated nitrogen at the opposite side of the alkyne linker was found to be relatively more stable. As a result, this cation was used for the following discussion (Table S1). Though these two isomers showed the same purplish‐blue fluorescence under ultraviolet excitation, they differed in fluorescence in the presence of an acid (Figure 1A–C). The Alk‐DQ‐1 showed a broad peak at 470–575 nm under blue excitation in the two‐dimensional fluorescence spectra, while the Alk‐DQ‐2 possessed a relatively narrow emission range of 450–500 nm under these conditions (Figure 1D). According to the color coordinates, the fluorescence color of protonated Alk‐DQ‐1 (Alk‐DQ‐1‐H^+^) should be defined as green (*x*=0.2369, *y*=0.4502) while the protonated Alk‐DQ‐2 (Alk‐DQ‐2‐H^+^) as greenish‐blue (*x*=0.1765, *y*=0.2743; Figure S5).

**Scheme 1:**
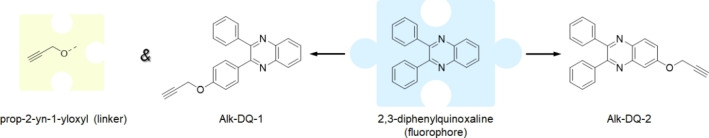
Chemical structure of positional isomers of 2,3‐diphenylquinoxaline with a prop‐2‐yn‐1‐yloxyl linker Alk‐DQ‐1 and Alk‐DQ‐2.

**Figure 1:**
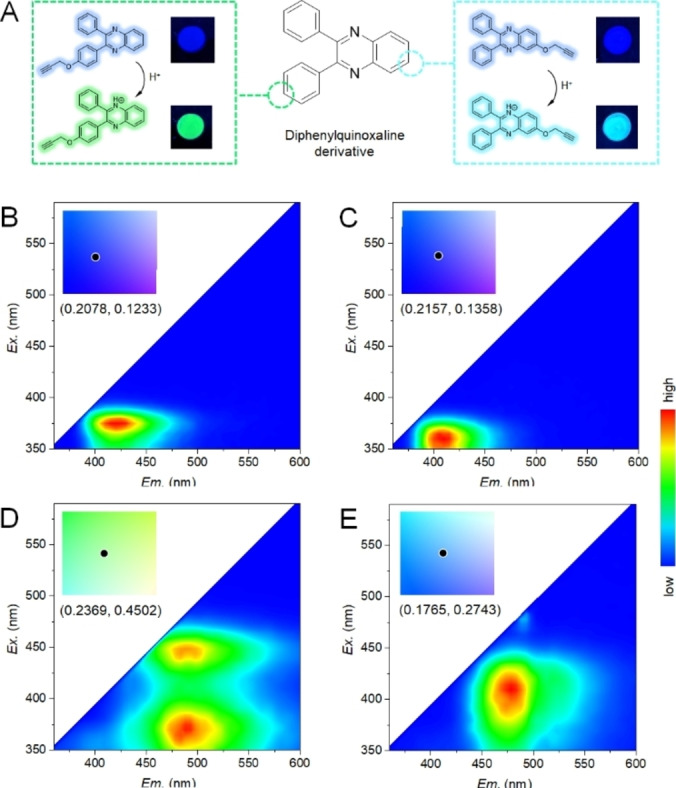
Fluorescence properties of Alk‐DQ‐1 and Alk‐DQ‐2. A) Change in structure and fluorescence of the two isomers in the presence of an acid. 2D fluorescence spectra of B) Alk‐DQ‐1, C) Alk‐DQ‐2, D)Alk‐DQ‐1‐H^+^, and E) Alk‐DQ‐2‐H^+^ in solution. Corresponding color coordinates were calculated and inserted according to the Commission internationale de l′éclairage (CIE) standard.

To better understand their acid‐responsive fluorescence, we compared their fluorescence in solution and on solid surface, before and after protonation. It was observed that the neutral fluorophores both in solution and on solid surface show similar emission in the deep blue range, but those on solid surface have a wider excitation range (Figures 1A, B and S6). This could be caused by the aggregation of fluorophores due to intermolecular interactions and the resulting splitting of absorption transitions. Owing to their fully conjugated structures, the fluorophores may form both H‐ and J‐type aggregates through different conformations, exhibiting both blue‐ and red‐shifted absorbance, similar to other conjugated organic fluorophores. However, the aggregation showed limited influence on the fluorescence properties for protonated fluorophores, where only a ∼5 nm red‐shift was observed in the excitation range. This phenomenon could be explained by the protonation‐induced electrostatic repulsion between the fluorophore molecules. Due to the strong electrostatic repulsion in the protonated quinoxaline moiety, the formation of aggregates is hindered, especially the H aggregates as the fluorophores are not planar. Therefore, according to the 2D fluorescence results, the obvious red‐shifted excitation and emission would be a result of protonation‐induced intermolecular charge redistribution as opposed to aggregation.

We subsequently simulated the electron distribution of both fluorophores before and after protonation. As displayed in the density functional theory (DFT) calculation results (Figure 2), the highest occupied molecular orbital (HOMO) located on the whole diphenylquinoxaline moiety in both Alk‐DQ‐1 and Alk‐DQ‐2, showed a symmetric distribution, indicating a fully conjugated structure. The electrons shifted to the quinoxaline moiety in the lowest unoccupied molecular orbital (LUMO), due to its relatively strong electron‐withdrawing nature, resulting in an asymmetric transition from HOMO to LUMO and contributing to the intramolecular charge transfer (ICT)‐induced emission. Though the prop‐2‐yn‐1‐yloxyl group violated the symmetric charge distribution in nonprotonated isomers, limited influence was observed on their band gaps as compared to the unmodified DQ fluorophore (Figure S7). After protonation, however, the influence of the prop‐2‐yn‐1‐yloxyl group on symmetry became significant. Due to nitrogen protonation, the electrons shifted from the quinoxaline to the attached benzene rings, increasing asymmetry of the electron distribution, promoting the ICT process, and thereby resulting in the narrowing of the band gap. The newly formed absorption peak in the visible range and red‐shifted fluorescence after acid treatment confirmed the narrowing band gap, indicating a reduced energy the for electron transition (Figure S8). Additionally, it was observed that the benzene ring on the same side as the protonated nitrogen generated more electrons in the LUMO state than the other in the HOMO state. As an electron‐donor, the prop‐2‐yn‐1‐yloxyl group had great impact on the shift of electrons and related ICT promotion, depending on its position in the structure. In Alk‐DQ‐1‐H^+^, the prop‐2‐yn‐1‐yloxyl group on the benzene ring served as a good electron donor, thereby enlarging the asymmetric distribution and assisting in the ICT promotion. On the contrary, the prop‐2‐yn‐1‐yloxyl group in Alk‐DQ‐2‐H^+^ led to a less efficient electron shift from the quinoxaline, which inhibited the promotion of ICT. When compared with the DQ‐H^+^ fluorophore without the alkyne linker, the Alk‐DQ‐1‐H^+^ had a much lower band gap, corresponding to a longer emission wavelength, while being higher than the band gap in Alk‐DQ‐2‐H^+^. In light of all these results, the different acid‐responsive fluorescence was attributed to the different electronic distribution in the protonated isomers induced by the position of the alkyne linker.

**Figure 2:**
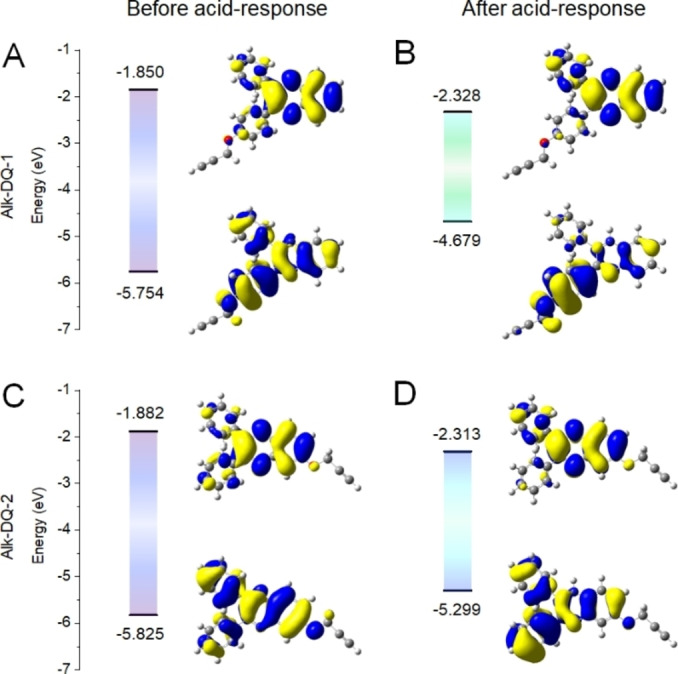
Mechanism of the differences before and after acid‐responsive fluorescence. Molecular orbital amplitude plots of the energy levels of A) Alk‐DQ‐1, B) Alk‐DQ‐1‐H^+^, C) Alk‐DQ‐2, and D) Alk‐DQ‐2‐H^+^. Calculations were performed at the B3LYP/6‐31G* level.

Notably, the protonation process was found to be reversible when a volatile acid (e. g., hydrogen chloride) was used for fumigation. The 2D fluorescence spectra showed that the newly formed emission peaks of the protonated fluorophore will gradually disappear as a function of standing time, while the original emission peak in the deep blue spectral range recovered (Figure S9). Due to their similar structures both isomers showed similar chemical reactivities and deprotonation time (Figure S10). As a result, the group of fluorophores, Alk‐DQ‐1 and Alk‐DQ‐2, could be used to construct multichannel and reversible fluorescent devices for encryption.

To clearly demonstrate these isomers practicability, fluorescent patterns were generated, based on both fluorophores using the combinatorial laser‐induced forward transfer (cLIFT) method (Figure 3A). In brief, pentafluorophenyl‐activated azidoacetic acid, embedded in a polymer, was laser transferred onto an amino‐functionalized acceptor slide in the desired patterns and formed amide bonds upon heating.^[8]^ The Cu^I^‐catalyzed click reaction was subsequently used to covalently attach the fluorophores to the azido‐modified surface patterns.^[9]^ Before the acidic treatment, only unrecognizably low signal and background noise were collected in the green (G) and red (R) channels, and were attributed to the scattered excitation light (Figure 3B), which confirmed that the information was well encrypted. After decryption by rapid acid fumigation, the pattern was clearly observed in the G channel and a fraction was obtained from the R channel (Figure 3C). This result was in agreement with the as‐designed image, suggesting that acid‐induced decryption was able to activate the fluorescence signal for the information readout (Figure S11). By subtracting the signals in different channels, new fragments were obtained in the newly defined G‐R subtraction channel. The above images, along with the corresponding signal distribution, indicated that the information can be encrypted separately in different fluorescence channels, to be used as a multichannel encryption device. Apart from image patterns, a similar protocol could be used to encrypt a variety of information types, such as characters, QR codes, and binary code patterns (Figure 3D). Thus, it is feasible to construct different encryption devices with this method.

**Figure 3:**
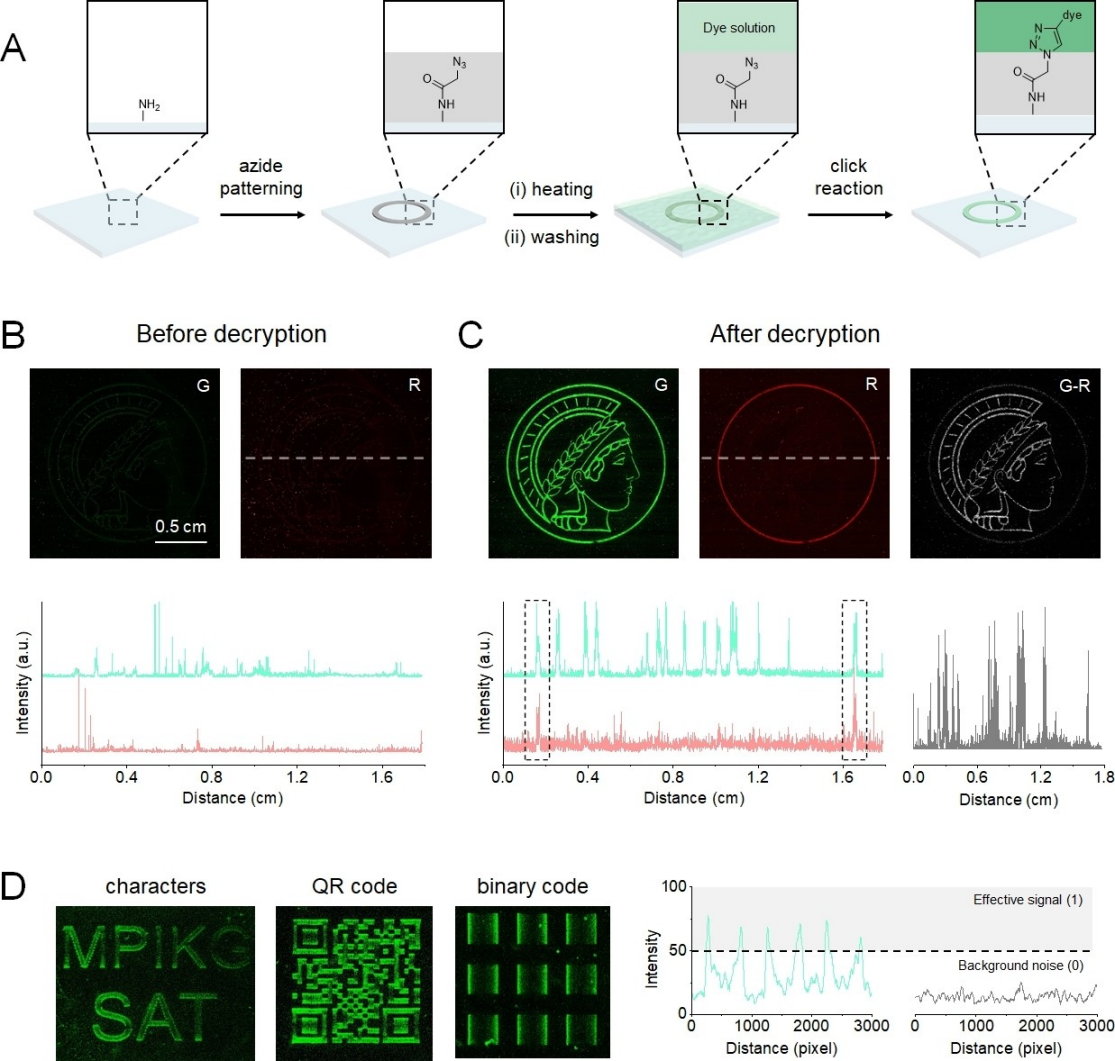
Multichannel fluorescent devices for encryption. A) Schematic illustration of the construction of chemically bound fluorescent patterns. Fluorescence image (top) and signal distribution (bottom) of an image pattern B) before and C) after decryption. Signal distribution paths are indicated in the fluorescence images by a dotted line. D) Fluorescence image of characters, QR code, and binary code patterns; right: decoded results of binary code.

To confirm that the use of positional isomers can improve the reliability, another fluorophore with a different structure, 2,3,5‐triphenyl‐6‐(4‐(prop‐2‐yn‐1‐yloxy)phenyl)pyrazine (Alk‐PZ), was synthesized and studied (Figures S12 and S13). All Alk‐DQ‐1, Alk‐DQ‐2, and Alk‐PZ showed signals in the G channel after acidic treatment. However, after standing at room temperature for few minutes, the signal of Alk‐PZ decreased more rapidly than the one for both Alk‐DQ‐1 and Alk‐DQ‐2 (Figure 4A). The relative fluorescence intensity (*I*/*I*_0_) of Alk‐PZ was significantly lower than Alk‐DQ at both 5 and 10 min (***p<0.001). In contrast, no statistic difference of *I*/*I*_0_ was observed between Alk‐DQ‐1 and Alk‐DQ‐2 (*p*>0.05). When the receiver operating characteristic curve analysis was used to quantify the distinguishability,^[10]^ the curves showed that it was sufficient to distinguish the spots of Alk‐DQ‐1 and Alk‐PZ using the difference of intensity over studied time with a precision rate of 98.4–100.0 % (Figure 4B). In contrast, the difference between Alk‐DQ‐1 and Alk‐DQ‐2 only allowed 65.6–67.2 % accuracy in predicting the spots, which was similar to the random prediction (50 %). Based on the above results, the difference of fluorescence change between Alk‐DQ‐1 and Alk‐PZ can be easily distinguished at various time points in the deprotonation process, but was relatively difficult for the group of Alk‐DQ‐1 and Alk‐DQ‐2. Therefore, it was reasonable to conclude that the multichannel encryption devices based on fluorescent positional isomers was much more reliable than those containing different types of fluorophores.

**Figure 4:**
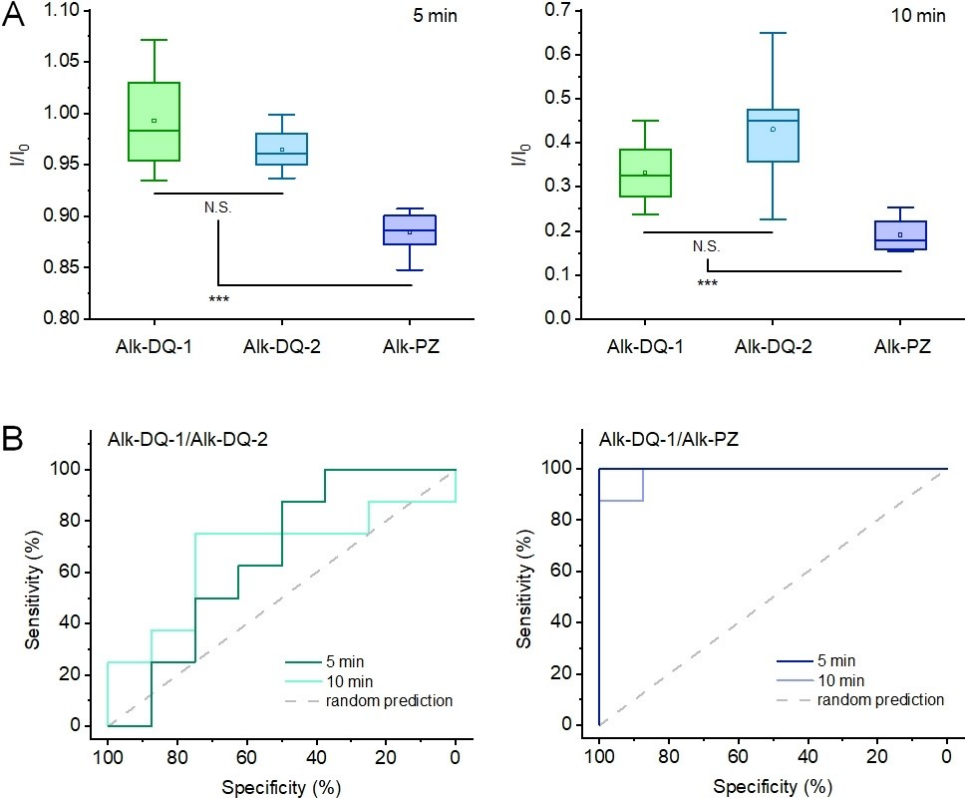
Quantification of reliability. A) Relative fluorescence intensity (*I*/*I*_0_) of patterns containing Alk‐DQ‐1, Alk‐DQ‐2, or Alk‐PZ in the green channel after 5 or 10 min of standing time. The starting fluorescence intensity (*I*_0_) was used as reference for normalization. B) Receiver‐operating characteristic curves presenting the probability of distinguishing directly between patterns containing different dyes, based on the signal difference as a function of time. (N.S. not significant, *p* >0.05; *** *p* <0.001).

In summary, multichannel encryption devices have been constructed based on fluorescent positional isomers with structural and reactive similarities. The small differences at the molecular level reduced the risk of untargeted (brute‐force) cracking and thereby resulted in a more reliable chemical encryption. This method will not only encourage the exploration of advanced optical materials for encryption purposes, but will bring a new approach to the field of chemical encryption.

## Conflict of interest

The authors declare no conflict of interest.

## Supporting information

As a service to our authors and readers, this journal provides supporting information supplied by the authors. Such materials are peer reviewed and may be re‐organized for online delivery, but are not copy‐edited or typeset. Technical support issues arising from supporting information (other than missing files) should be addressed to the authors.

Supporting Information
