# Supplementary material for: Position Matters: Fluorescent Positional Isomers for Reliable Multichannel Encryption Devices
Source: Chemistry. 2021 Oct 29;27(65):16098–102. doi: 10.1002/chem.202103441 (PMC9298033; doi:10.1002/chem.202103441)
Supplement: Supplementary file 1 — Supporting Information [file CHEM-27-16098-s001.pdf]

Chemistry–A European Journal

Supporting Information

Position Matters: Fluorescent Positional Isomers for Reliable Multichannel Encryption Devices

Yuxin Liu, Peter H. Seeberger, Nabyl Merbouh,* and Felix F. Loeffler*

Experimental sections

1.1 Chemicals

The azidoacetic acid, *N*,*N'*-diisopropylcarbodiimide, pentafluorophenol, and piperidine were purchased from Acros Organics B.V.B.A. The polystyrene was purchased from Sigma-Aldrich. The dichloromethane (DCM), *N*,*N*-dimethylmethanamide (DMF), dimethyl sulfoxide (DMSO), and methanol were purchased from Thermo-Fisher Scientific International, Inc. All solvents were reagent grade and used without further purification. All synthetic reagents were purchased from Sigma Aldrich and used as is. The precursor 1-(4-hydroxyphenyl)-2-phenyl-1,2-ethanedione was synthesized from 1-(4-hydroxyphenyl)-2-phenylethanone and selenium dioxide according to the procedure of Zarghi *et al.*[1] while 1-phenyl-2-[4-(2-propyn-1-yloxy)phenyl]-1,2-ethanedione was synthesized according to Quin *et al.*[2] 4-(prop-2-yn-1-yloxy)benzene-1,2-diamine was synthesized according to Zhang *et al.*[3]

1.2 Characterization

^{1}H-NMR and ^{13}C-NMR spectra were recorded on an Avance III 400, Avance III 500 Brucker spectrometers. All NMR spectra were referenced to the residual chloroform ($CDCl_3$) resonance at 7.26 ppm for ^{1}H-NMR and 77.03 ppm for ^{13}C-NMR. High Resolution Mass Spectrometry was recorded on a 6210 ESI-TOF mass spectrometer (Agilent Technologies, USA).

1.3 General Synthetic Procedure.

A solution of benzil derivative (1 equiv.) and diamine (2.5 equiv.) in acetic acid was stirred under reflux overnight. The solvent was removed under vacuum and the crude product was purified by column chromatography on silica with using 20% ethyl acetate in hexanes as eluent to yield the desired product in yields ranging from 10 to 35% *(non optimized syntheses)*.

1.4 2-Phenyl-3-(4-(prop-2-yn-1-yloxy)phenyl)quinoxaline (Alk-DQ-1)

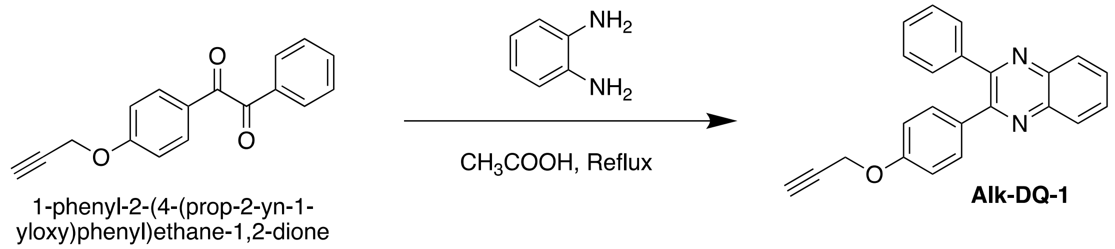

^{1}H-NMR (400 MHz, $CDCl_3$) δ 8.22 – 8.11 (m, 2H), 7.81 – 7.71 (m, 2H), 7.59 – 7.52 (m, 2H), 7.48 (d, J = 8.8 Hz, 2H), 7.43 – 7.33 (m, 3H), 6.93 (d, J = 8.8 Hz, 2H), 4.71 (d, J = 2.4 Hz, 2H), 2.53 (t, J = 2.4 Hz, 1H) ppm. ^{13}C-NMR (101 MHz, $CDCl_3$) δ 55.85, 75.80, 78.23, 114.69, 128.40, 128.86, 129.08, 129.18, 129.78, 129.81, 130.01, 131.37, 132, 24, 139.24, 141.05, 141.25, 152.87, 153.44, 158.17 ppm. HRMS (ESI/TOF) m/z: $[M + H]^+$ Calcd for $C_{23}H_{17}N_2O$ 337.1335; Found 337.1349.

1.5 2,3-Diphenyl-6-(prop-2-yn-1-yloxy)quinoxaline (Alk-DQ-2)

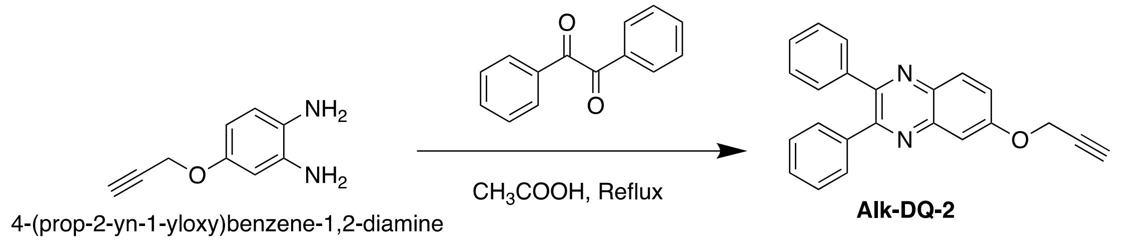

^{1}H-NMR (400 MHz, $CDCl_3$) δ 8.09 (d, J = 9.2 Hz, 1H), 7.59 (d, J = 2.8 Hz, 1H), 7.56 – 7.42 (m, 5H), 7.40 – 7.30 (m, 6H), 4.88 (d, J = 2.4 Hz, 2H), 2.59 (t, J = 2.4 Hz, 1H) ppm. ^{13}C-NMR (101 MHz, $CDCl_3$) δ 56.27, 76.32, 77.67, 107.83, 123.46, 128.31, 128.34, 128.65, 128.87, 129.83, 129.85, 130.43, 137.64, 139.00, 142.36, 151.41, 153.51, 158.69 ppm. HRMS (ESI/TOF) m/z: $[M + H]^+$ Calcd for $C_{23}H_{17}N_2O$ 337.1335; Found 337.1350.

1.6 2,3,5-Triphenyl-6-(4-(prop-2-yn-1-yloxy)phenyl)pyrazine (Alk-PZ)

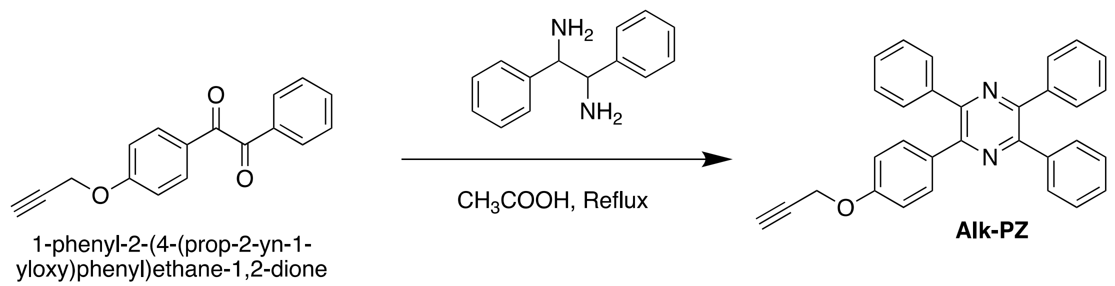

^{1}H-NMR (400 MHz, $CDCl_3$) δ 7.71-7.62 (m, 8H), 7.38-7.31(m, 9H), 6.95 (d, J = 9 Hz, 2H), 4.73 (d, J = 2.4 Hz, 2H), 2.56 (t, J = 2.4 Hz, 1H) ppm. ^{13}C-NMR (101 MHz, $CDCl_3$) δ 55.85, 75.67, 78.36, 114.64, 128.22, 128.29, 128.53, 128.57, 129.80, 129.86, 129.88, 131.25, 131.70, 138.53, 138.56, 138.71, 147.78, 147.99, 148.01, 148.27, 158.06 ppm. HRMS (ESI/TOF) m/z: $[M + H]^+$

Calcd for $C_{31}H_{23}N_2O$ 439.1805; Found 439.1812.

1.7 Preparation of the donor and acceptor slides

Preparation of spin-coating solution: In a typical experiment, 81.0 mg of polystyrene was previously dissolved in 1350 μL DCM. Then, 7 μL of azidoacetic acid was mixed with 143 μL DMF under vortex, with the addition of 13.8 μL *N,N'*-diisopropylcarbodiimide and 16.4 mg pentafluorophenol in order. After vortex for 0.5 min and spin down with mini centrifuge, 150 μL of the activated azidoacetic acid solution was mixed with polystyrene solution. This prescription was for 3 donor slides and the solution should be freshly prepared just before use.

Preparation of donor slide: The blank donor slides were prepared by covering microscope glass slides with self-adhesive polyimide foil (Kapton HN, DuPont, 25 μm polyimide layer with a 45 μm siloxane-based adhesive layer; CMC Klebetechnik). Then, the as-prepared solution was spin-coated on the blank donor slides (500 μL per slide, 80 rounds per second). The donor slides would be dried in a jet of air before use.

Preparation of acceptor slide: PEGMA-co-MMA (PEPperPRINT GmbH) slides with a terminal *N*-[(9H-Fluoren-9-ylmethoxy)carbonyl] (Fmoc)-protected β-alanine were used as the acceptor slides with the following treatments. The PEGMA-co-MMA slides were warmed to room temperature under nitrogen protection, then transferred to a clean petri dish, and vibrated on an orbital shaker. First, the acceptor slides were pre-swelled by immersing them in DMF for 20 min. Subsequently, for Fmoc-deprotection the acceptor slides were immersed in 20 % [v/v] piperidine in DMF for 20 min. Finally, the acceptor slides were washed with DMF (3 x 3 min), methanol (1 x 2 min), and DCM (1 x 1 min), then dried in a jet of air. The acceptor slides would be used as soon as possible after the above treatments.

1.8 Preparation of the fluorescent patterns

Parameters for pattern printing: The image pattern was printed by our cLIFT laser system, as described in our previous reports.[4] 60 % laser power was applied for the printing with a pulse duration of 6 ms. The characters, QR code, and binary patterns were printed by another LIFT system, described in our previous reports.[5] 50 % laser power was applied for the printing at a speed of 1000 mm min^{-1}. For filling solid patterns with lines, the interval between lines was 0.1 mm.

Modifying patterns with different fluorophores: Cu-catalyzed click reaction was used to chemically bind fluorophores on azido-contained patterns.[4] In a typical experiment, 5.3 mg $CuSO_4$ and 10.0

mg sodium ascorbate were added to a mixture of 300 μL DMSO and 100 μL water in a vial. The vial was shaken for 5 min and, afterwards, the precipitate was centrifuged and the remaining solution was passed through a syringe filter (0.2 mm, polypropylene). Then, 3.0 mg Alk-DQ-1 was dissolved in the solution and then applied on the patterned acceptor surface. The prepared solution was poured between the acceptor slide and another clean glass slide, then shaking overnight in the dark. The acceptor slides were washed with DMSO (1 x 10 min) and water (1 x 30 min). The clicking of Alk-DQ-2 was then performed in the same process by repeating both, pattern printing and Cu-catalyzed click reaction (Figure S8). For the Alk-PZ, 3.7 mg solid powder was used to prepare the solution. The fluorescent patterns are treated with the vapor of hydrogen chloride before fluorescence imaging. A filter paper absorbed with concentrated hydrogen chloride solution was used to slow down the deprotonation process by providing continuous acid vapor presence.

1.9 Determination of 2D fluorescence spectra

The 2D fluorescence spectra were performed on a Jasco FP-8300 fluorescence spectrometer with a xenon lamp light source. For the determination in solution, the fluorophores were dissolved in DCM and protonated by concentrated hydrogen chloride solution (~37 wt%). For the determination on solid surface, small pieces of filter paper absorbed with concentrated hydrogen chloride solution were stuck on the glass slide to slow down the deprotonation process by providing continuous acid vapor presence.

1.10 Statistical analysis

The statistic test was two-sided. Receiver-operating characteristic curve analysis was utilized to determine the distinguishability between signal intensities of different fluorophores in green channel.[6] The receiver-operating characteristic curve analysis captured the trade-off between sensitivity and specificity, while changing a discrimination threshold, but it was summarized as a single measurement. The sensitivity (true positive rate) was plotted against the specificity (true negative rate) in the characteristic curve as a function of a variety of thresholds of class prediction probabilities. The overall accuracy depended on the overlap of the output distributions for the two classes, which were the pair of fluorophores in this case. Values would range between 0.5 and 1.0, where a value of 0.5 indicated that the two distributions were identical while a value of 1.0 indicated that they were completely orthogonal. The area under curve was used as a lone measure to evaluate the efficiency of identification according to the probability assigned to the positive class. It was

calculated by the trapezoidal method of integration with the corresponding confidence intervals of 95 %. In this work, the randomized prediction, with the area under curve of 0.5, was used as the reference to optimize the minimal difference.

[1] A. Zarghi, S. Arfaei, F. H. Shirazi, *Med. Chem. Res.* **2013**, *22*, 3897-3904.

[2] H.-T. Qin, X. Xu, F. Liu, *ChemCatChem* **2017**, *9*, 1409-1412.

[3] P. Zhang, Y. Tian, H. Liu, J. Ren, H. Wang, R. Zeng, Y. Long, J. Chen, *Chem. Commun.* **2018**, *54*, 7231-7234.

[4] M. Mende, A. Tsouka, J. Heidepriem, G. Paris, D. S. Mattes, S. Eickelmann, V. Bordoni, R. Wawrzinek, F. F. Fuchsberger, P. H. Seeberger, C. Rademacher, M. Delbianco, A. Mallagaray, F. F. Loeffler, *Chem.-Eur. J.* **2020**, *26*, 9954-9963.

[5] S. Eickelmann, A. Tsouka, J. Heidepriem, G. Paris, J. F. Zhang, V. Molinari, M. Mende, F. F. Loeffler, *Adv. Mater. Technol.* **2019**, *4*, 1900503.

[6] Y. X. Liu, Z. Wei, J. Zhou, Z. F. Ma, *Nat. Commun.* **2019**, *10*, 5361.

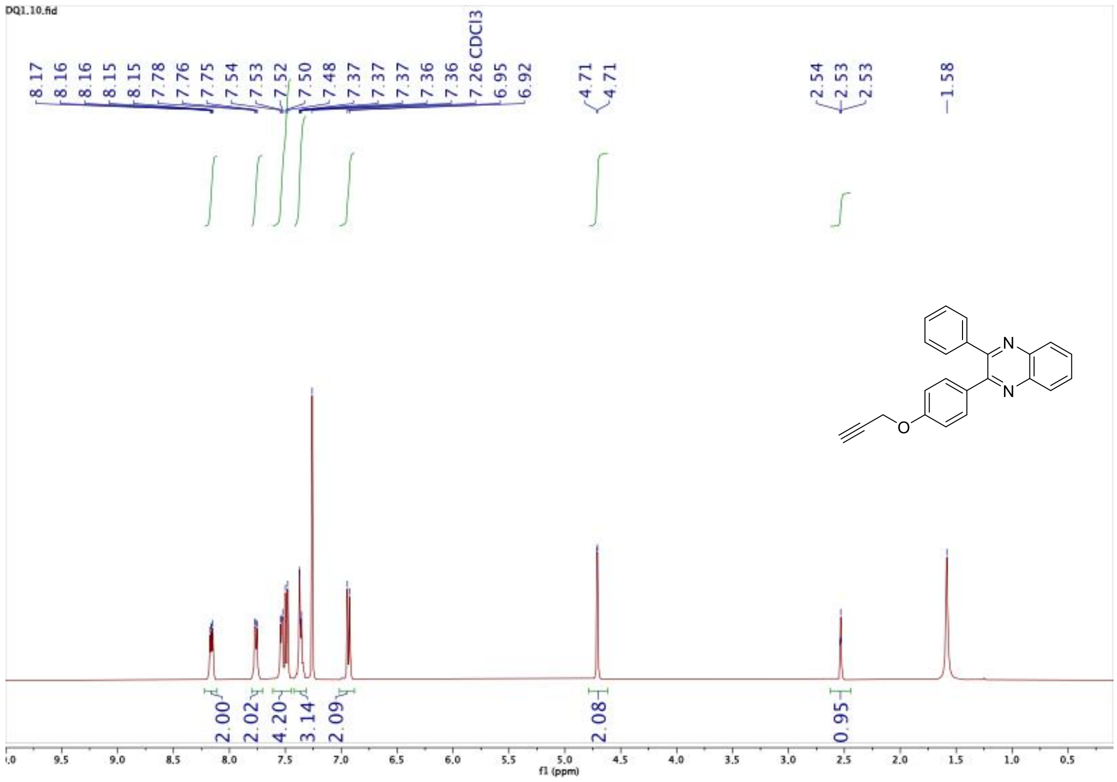

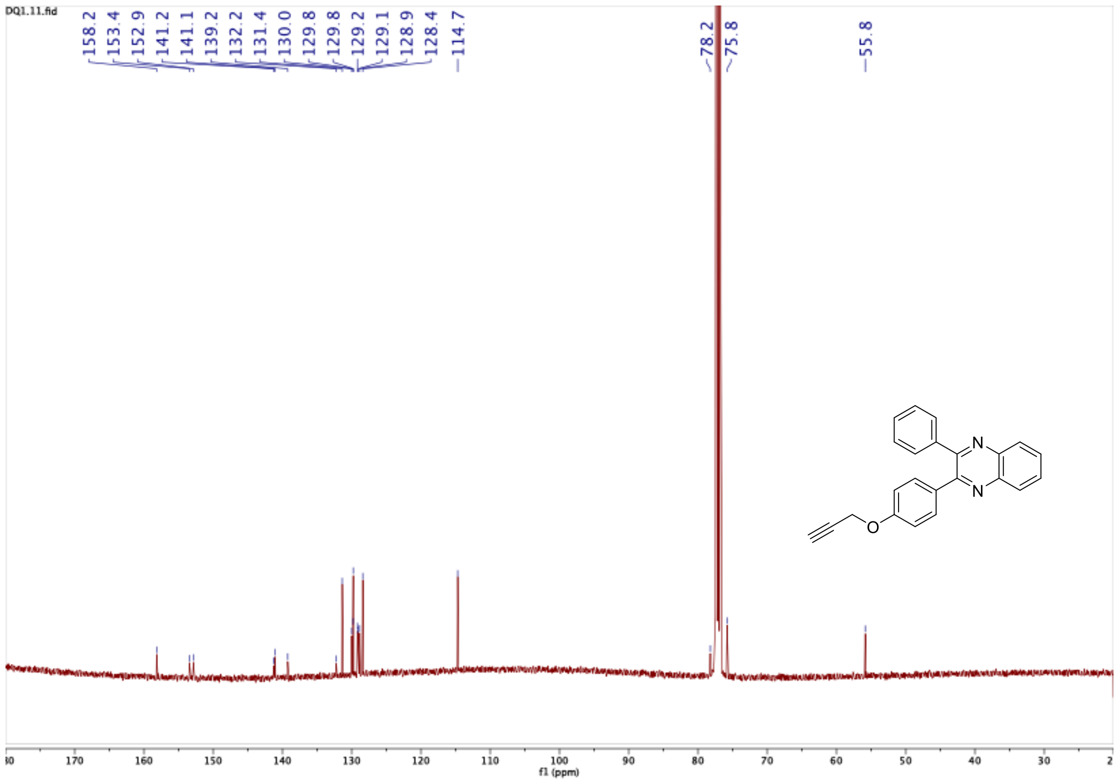

Figure S1. ^{1}H and ^{13}C NMR spectra of the Alk-DQ-1.

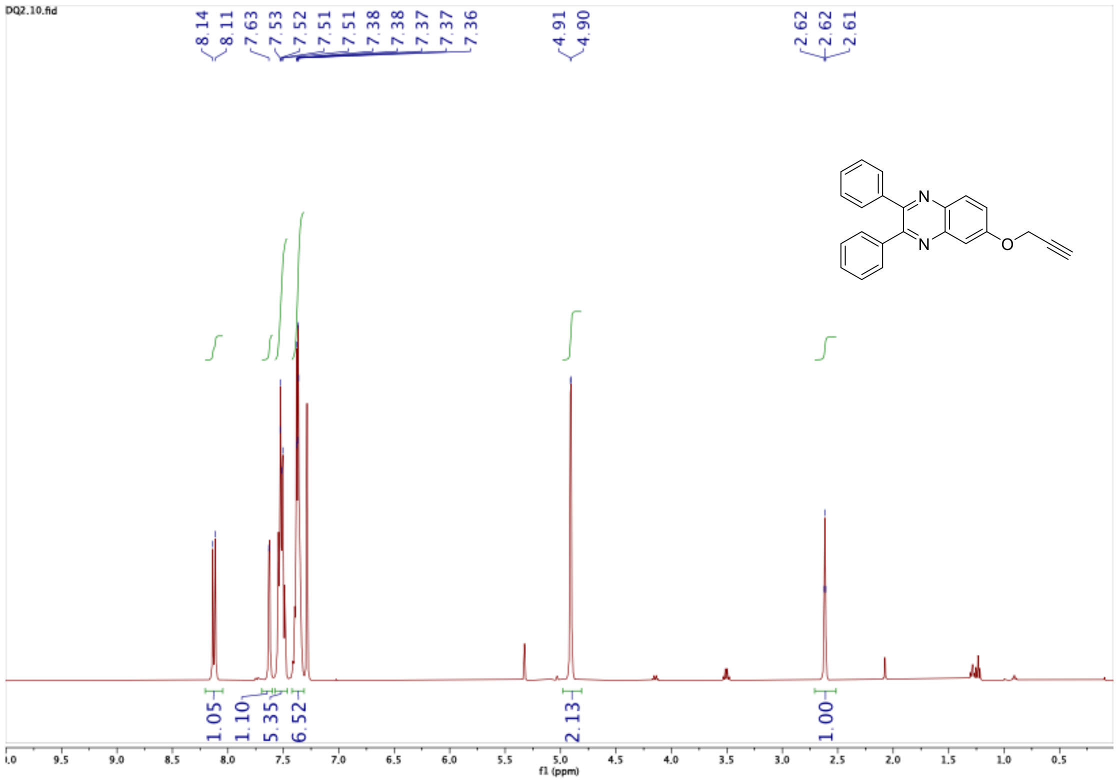

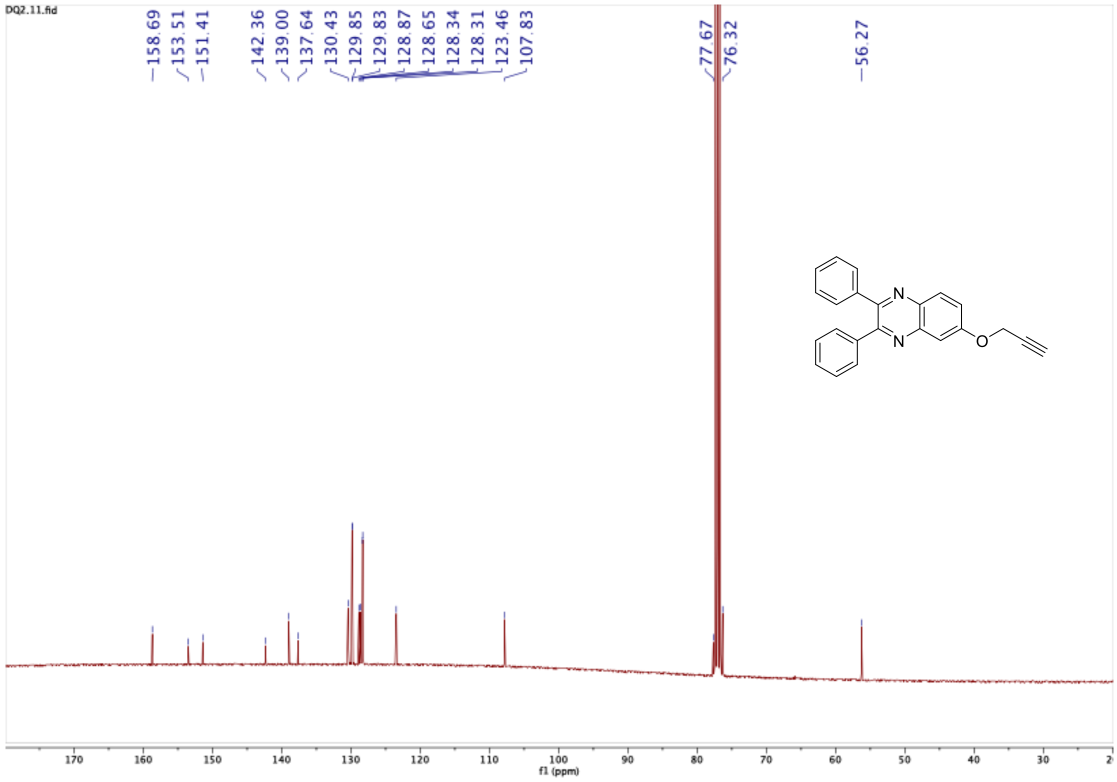

Figure S2. ^{1}H and ^{13}C NMR spectra of the Alk-DQ-2.

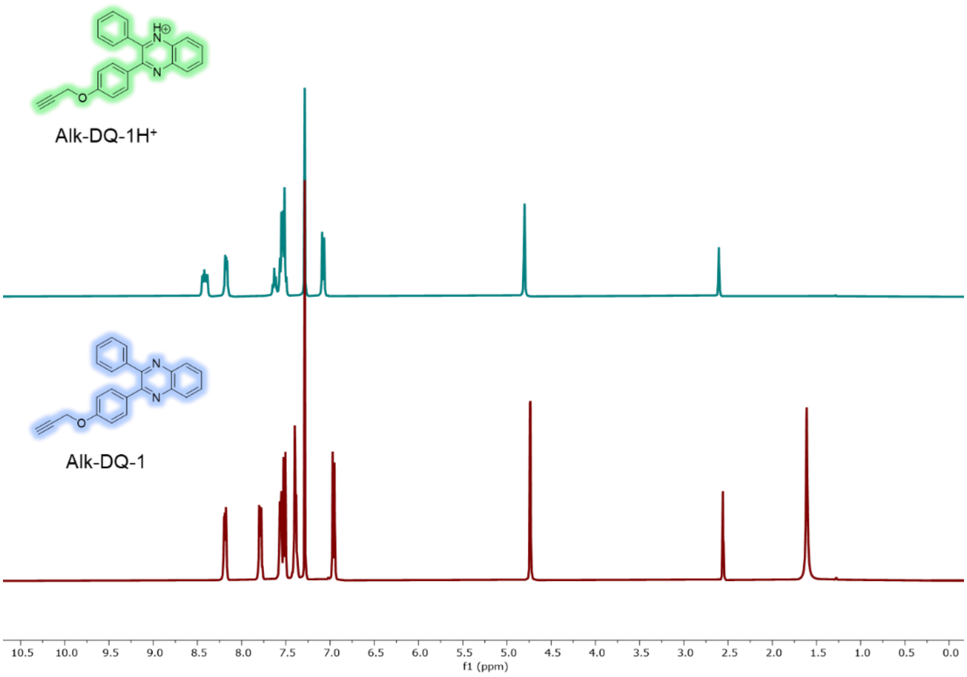

Figure S3. ^{1}H spectra of the Alk-DQ-1H^{+}. ^{1}H spectra of the Alk-DQ-1 in Figure S1 was included for comparison.

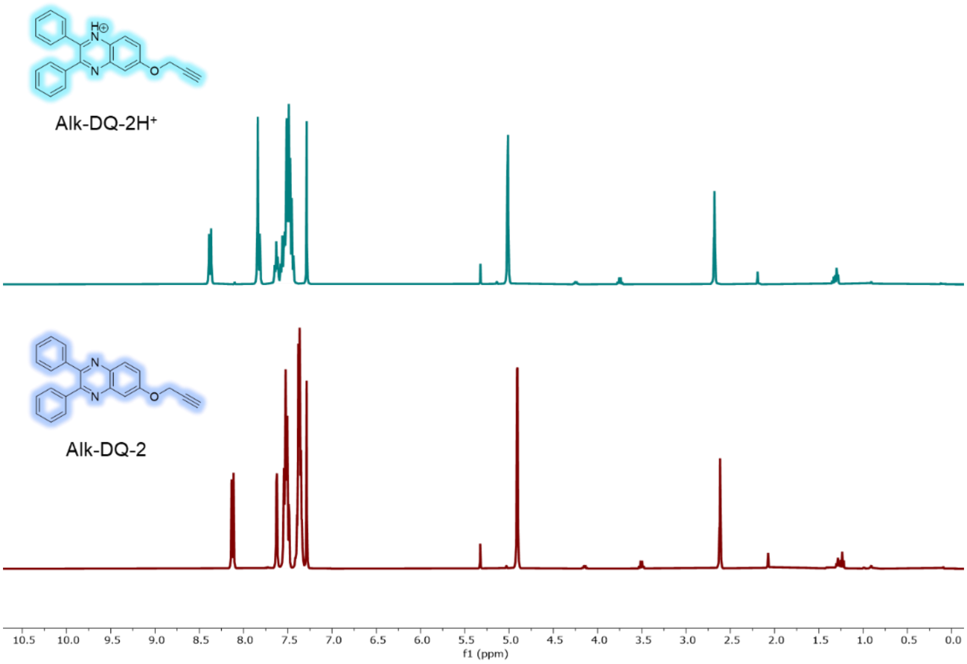

Figure S4. ^1H spectra of the Alk-DQ-2H$^+$. ^1H spectra of the Alk-DQ-2 in Figure S2 was included for comparison.

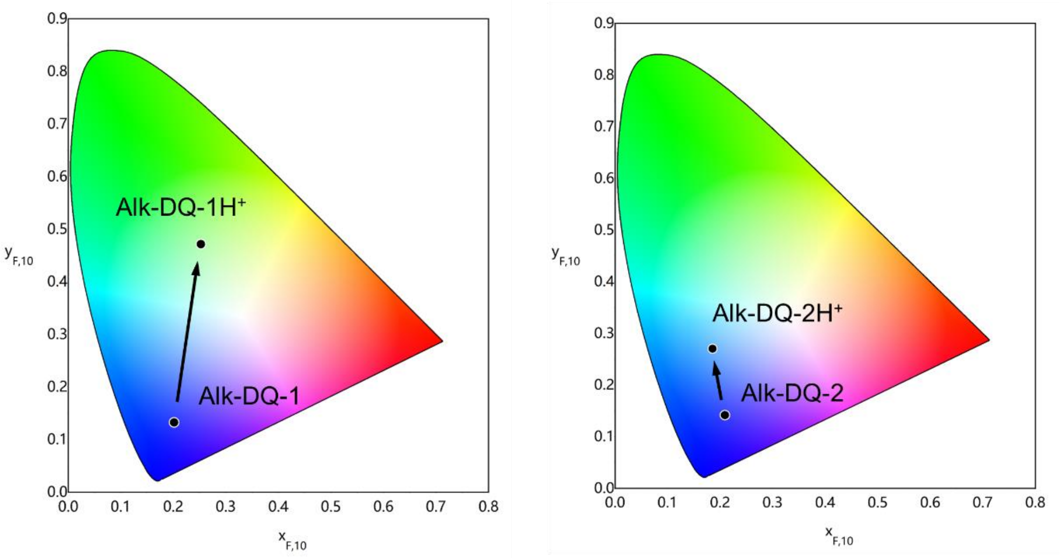

Figure S5. Commission internationale de l'éclairage (CIE) image of color coordinates.

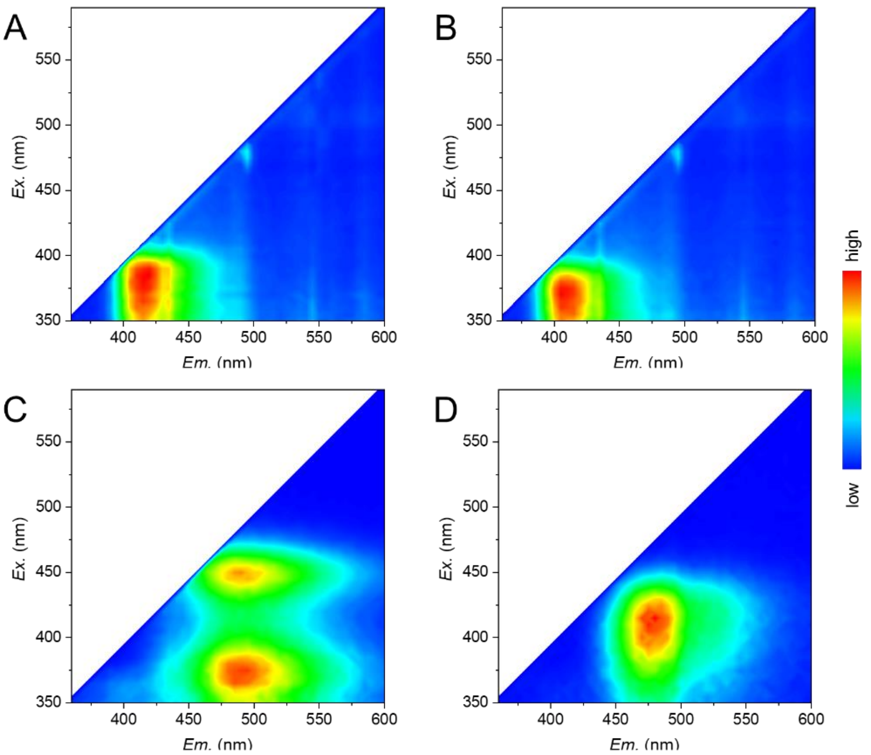

Figure S6. 2D fluorescence spectra of Alk-DQ-1 A), Alk-DQ-2 B), Alk-DQ-1-H^+ C), and Alk-DQ-2-H^+ D) on solid surface.

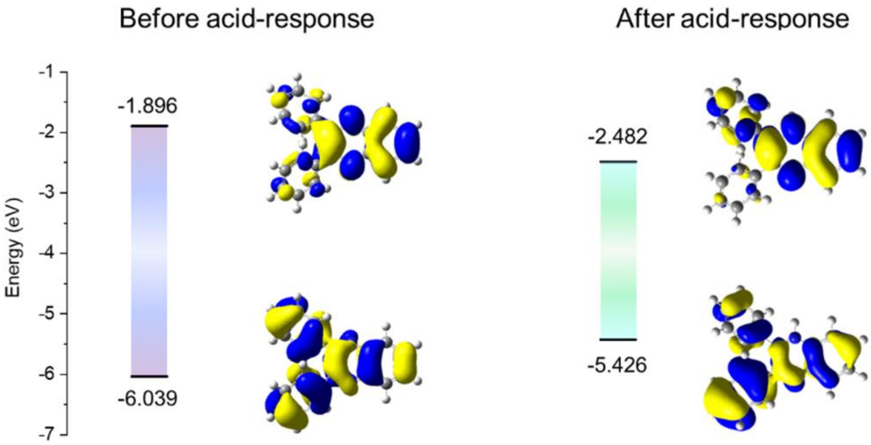

Figure S7. Molecular orbital amplitude plots of the energy levels of non-modified DQ before and after acid treatment. Calculations were performed at the B3LYP/6–31G* level.

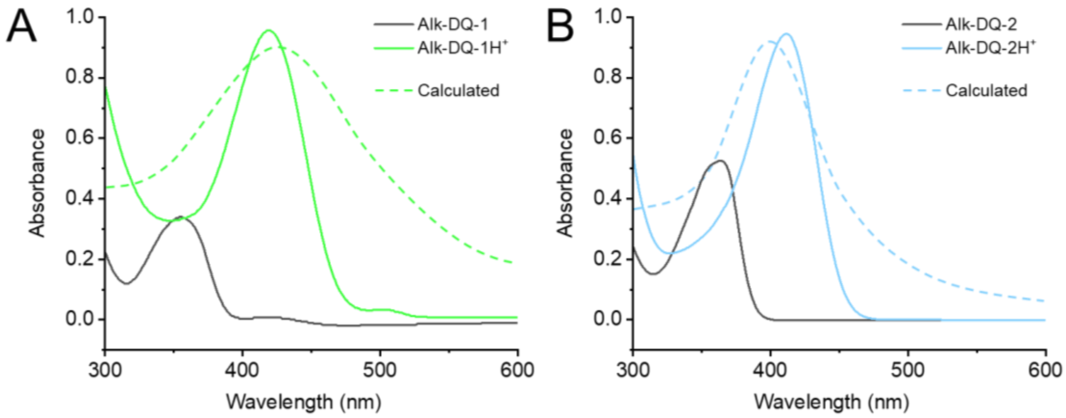

Figure S8. Absorbance spectra of Alk-DQ-1 A) and Alk-DQ-2 B) before and after protonation. The calculated results were included.

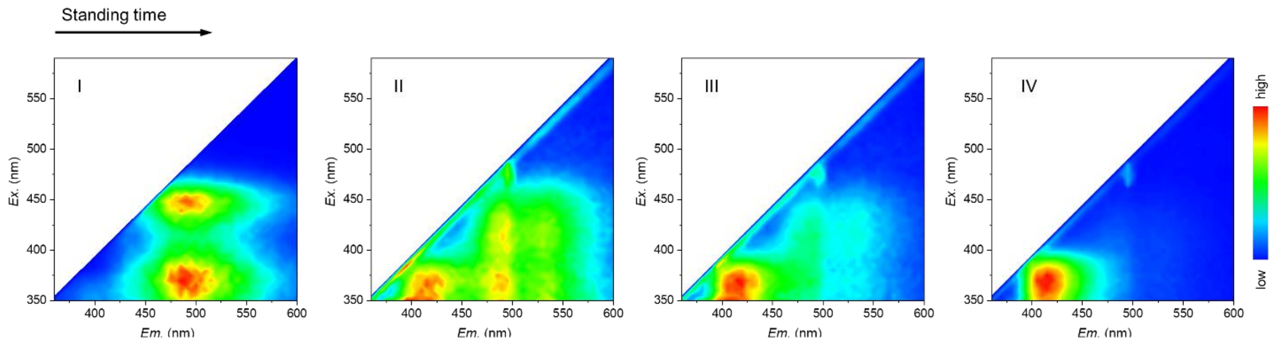

Figure S9. 2D fluorescence spectra of Alk-DQ-1-H^+ on solid surface after 0 (I), 5 (II), 10 (III), and 20 min (IV). The first spectrum was obtained by sticking a small piece of filter paper absorbed with concentrated hydrochloric acid. The second to fourth spectrum were obtained at different time points after removing the filter paper.

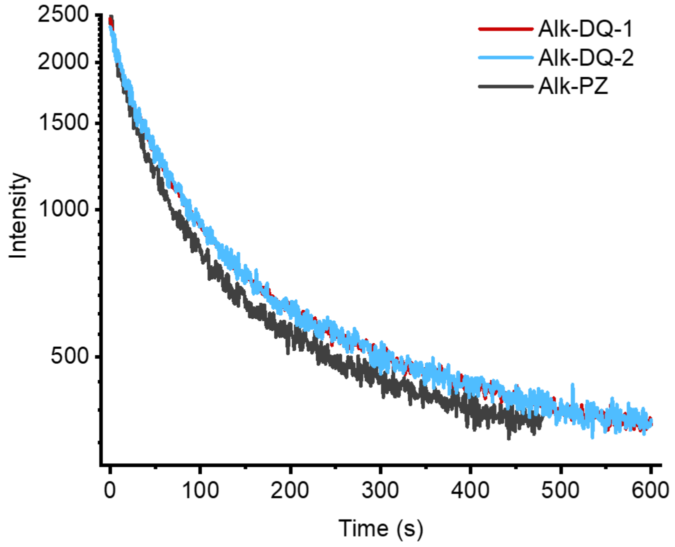

Figure S10. Dynamic fluorescent spectra of Alk-DQ-1, Alk-DQ-2, and Alk-PZ during the deprotonation process.

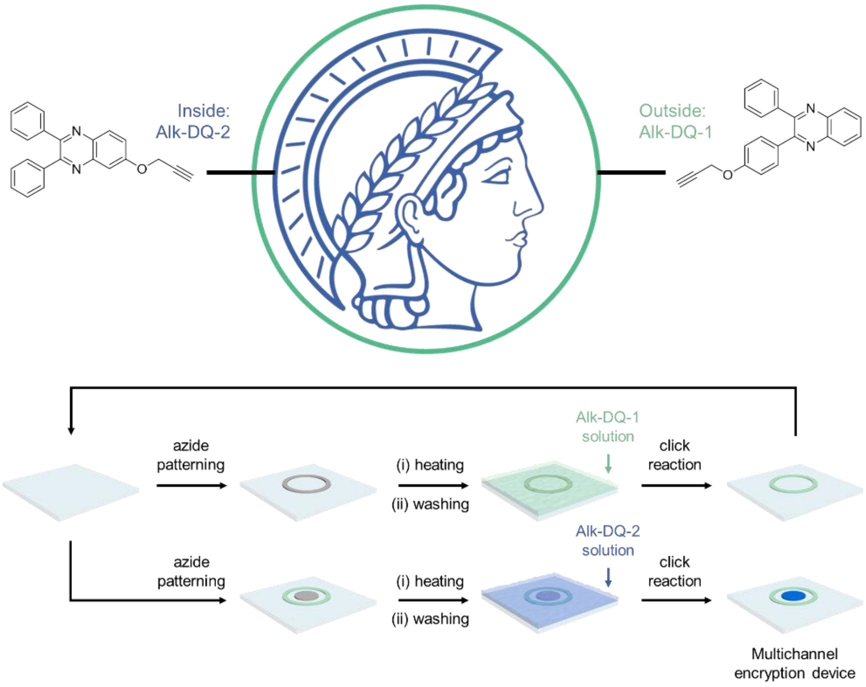

Figure S11. Schematic illustration of the design and preparation process of the as-presented image pattern.

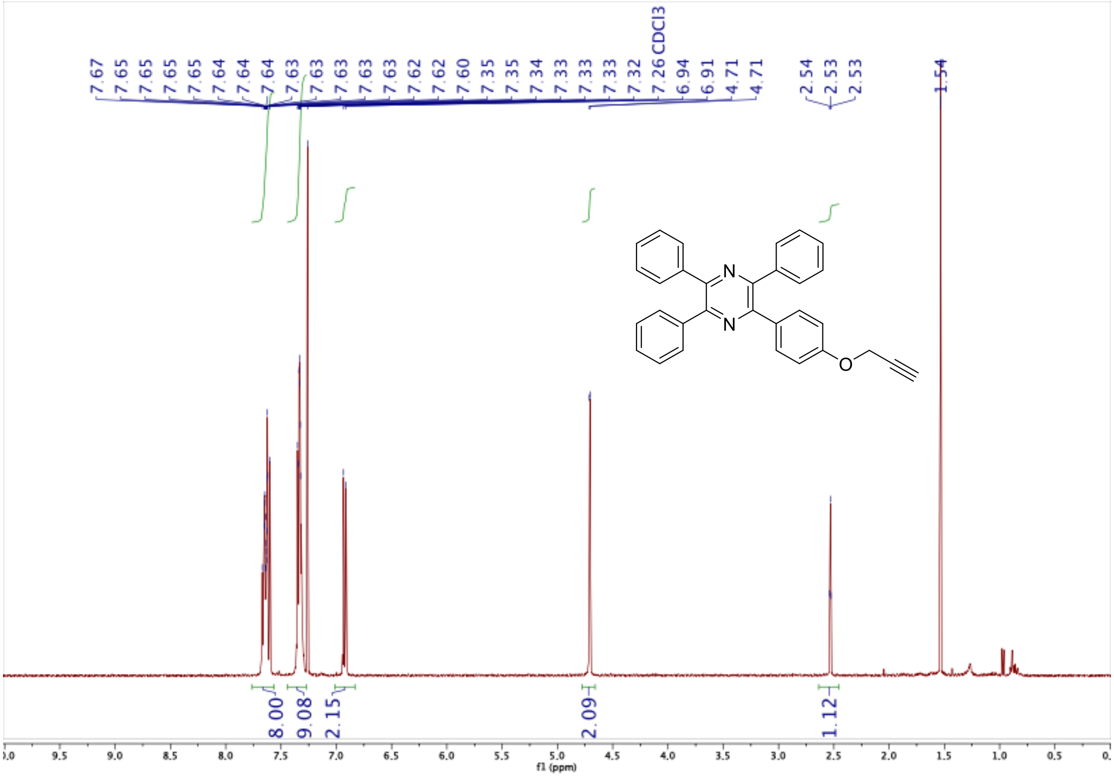

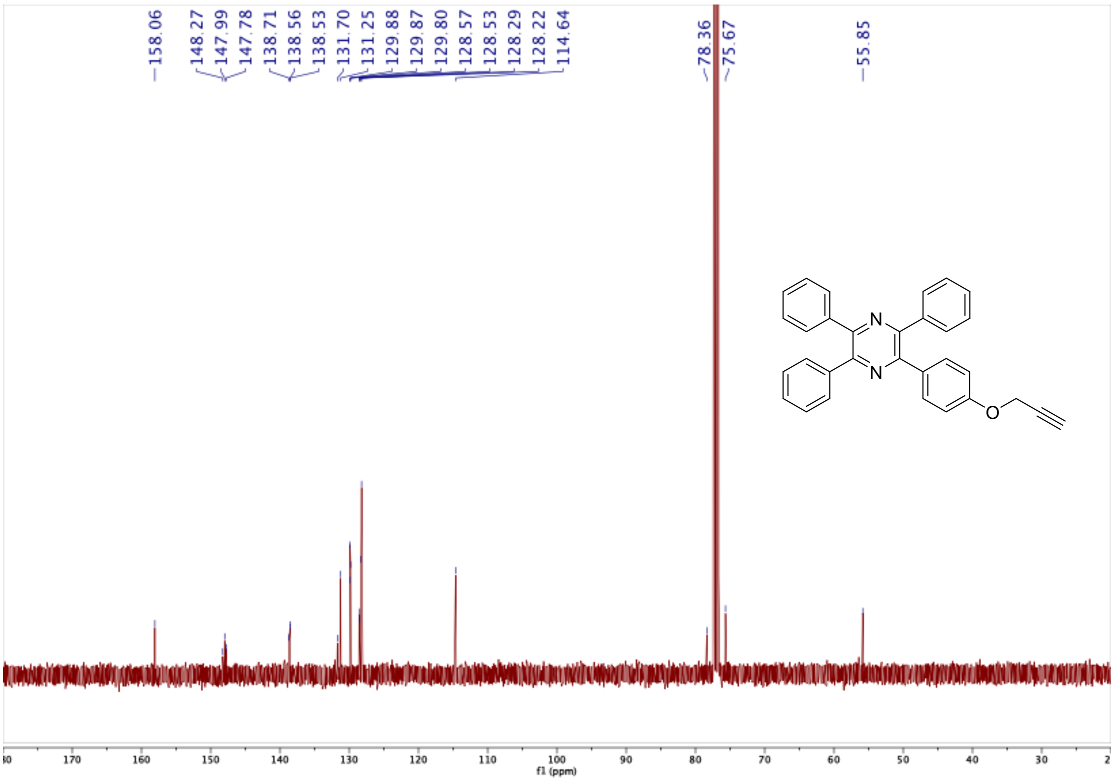

Figure S12. ^{1}H and ^{13}C NMR spectra of the Alk-PZ.

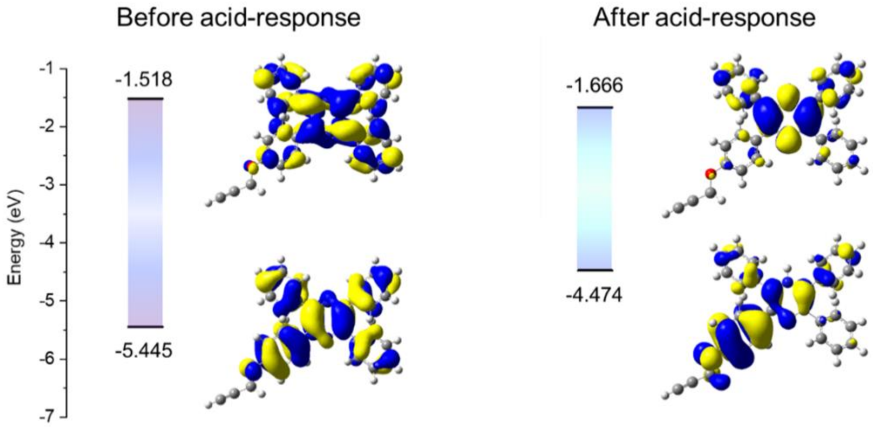

Figure S13. Molecular orbital amplitude plots of the energy levels of Alk-PZ before and after acid treatment. Calculations were performed at the B3LYP/6–31G* level.

Table S1. The energy of neutral Alk-DQ-1, Alk-DQ-2 and different monoprotonated forms.

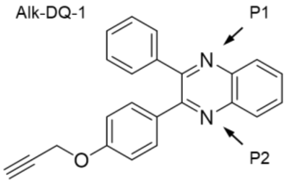

Neutral *E* (Hartree)	Cation	*E* (Hartree)	Energy difference (kJ mol^{-1})
-1070.8340	P1	-1071.2251	3.4132
	P2	-1071.2238	

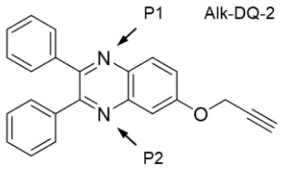

Neutral *E* (Hartree)	Cation	*E* (Hartree)	Energy difference (kJ mol^{-1})
-1070.8319	P1	-1071.2537	9.7144
	P2	-1071.2500	
